# Supplementary material for: Chromatin states shaped by an epigenetic code confer regenerative potential to the mouse liver
Source: Nat Commun. 2021 Jul 5;12:4110. doi: 10.1038/s41467-021-24466-1 (PMC8257577; doi:10.1038/s41467-021-24466-1)
Supplement: Supplementary file 1 — Supplementary Information [file 41467_2021_24466_MOESM1_ESM.pdf]

Suppl. Fig1

a

E-S1	E-S2	E-S3	E-S4	E-S5	E-S6	
0.2828	0.0158	0.0027	0.0043	0.0025	0.0030	E-POLR2A
0.9542	0.0145	0.0002	0.0003	0.0000	0.0025	E-H3K4me3
0.6606	0.4932	0.0032	0.0076	0.0005	0.0061	E-H3K27ac
0.3395	0.7234	0.0431	0.0206	0.0008	0.0279	E-H3K4me
0.3353	0.2854	0.1719	0.1052	0.0063	0.0368	E-H3K79me3
0.0406	0.1312	0.0546	0.7542	0.0008	0.0009	E-H3K36me3
0.1581	0.0235	0.0149	0.0177	0.0212	0.1855	E-H3K27me3

b

Genome Fraction (Encode data)

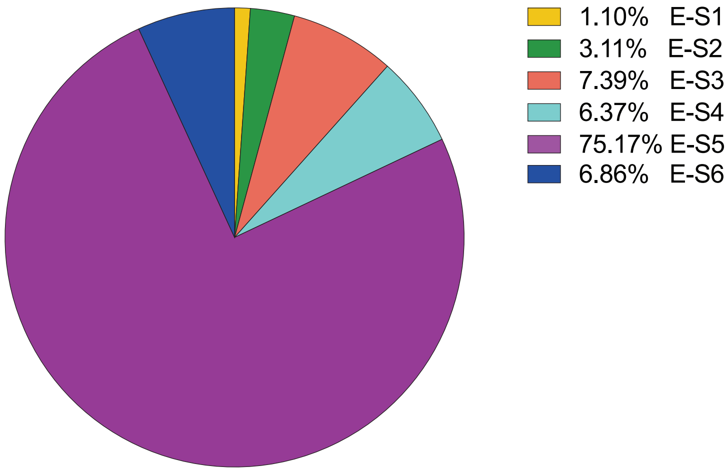

Supplementary Figure 1. Mouse ENCODE data defines 6 chromatin states in the liver
a. Heatmap of the emission parameters shows chromatin mark combinations associated with each state.
b. The fraction of mouse liver genome region shared by each state.

Suppl. Fig2

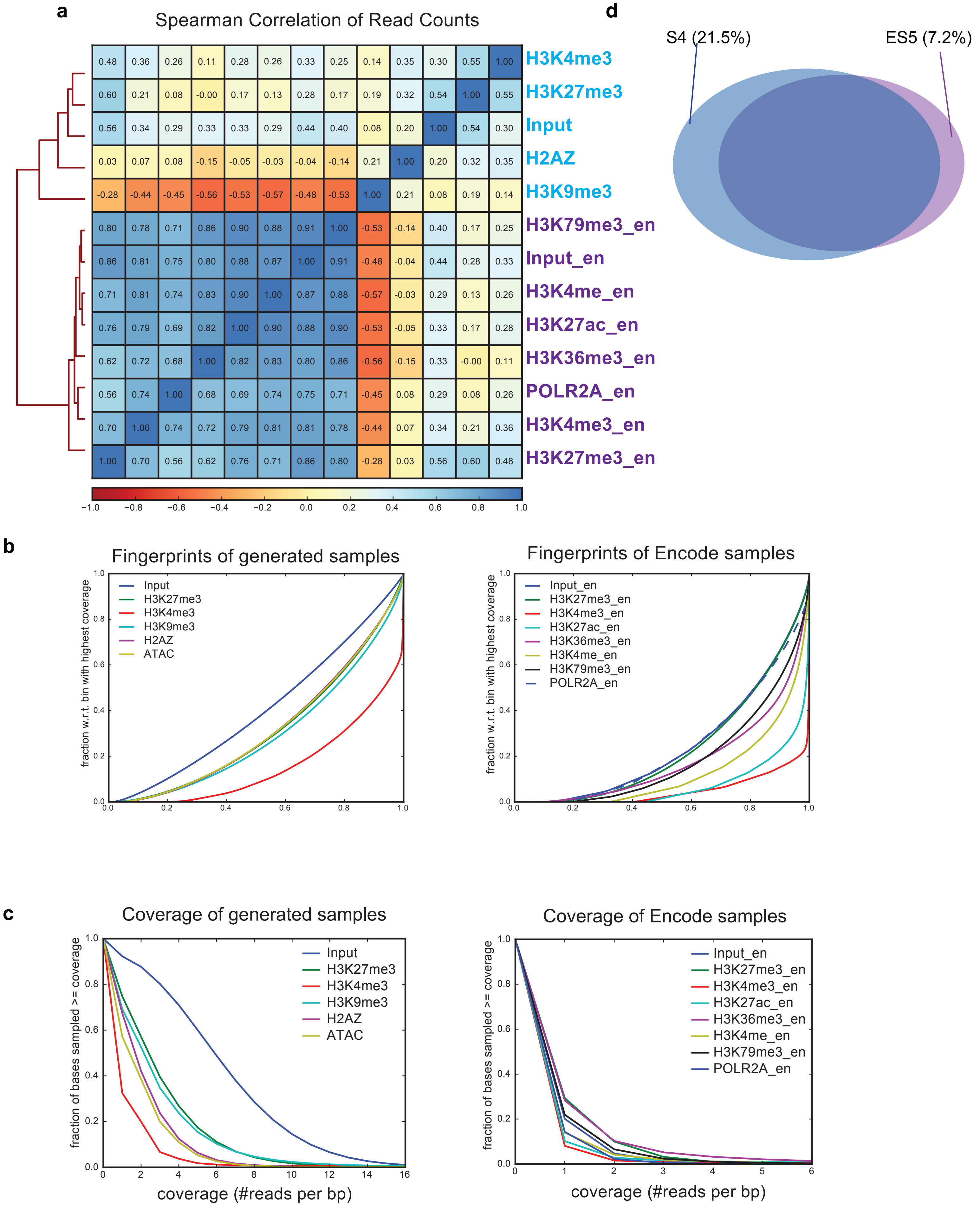

Supplementary Figure 2. Comparative analysis of in-house datasets and ENCODE datasets

a. Spearman correlation of histone markers compared to ENCODE datasets.

b. Fingerprints of in-house data set and ENCODE data set show enrichment quality of each markers.

c. Coverage comparison between the datasets generated in this study and ENCODE datasets.

d. Overlap of predicted empty states from two datasets cross validated the chromatin states prediction.

Suppl. Fig3

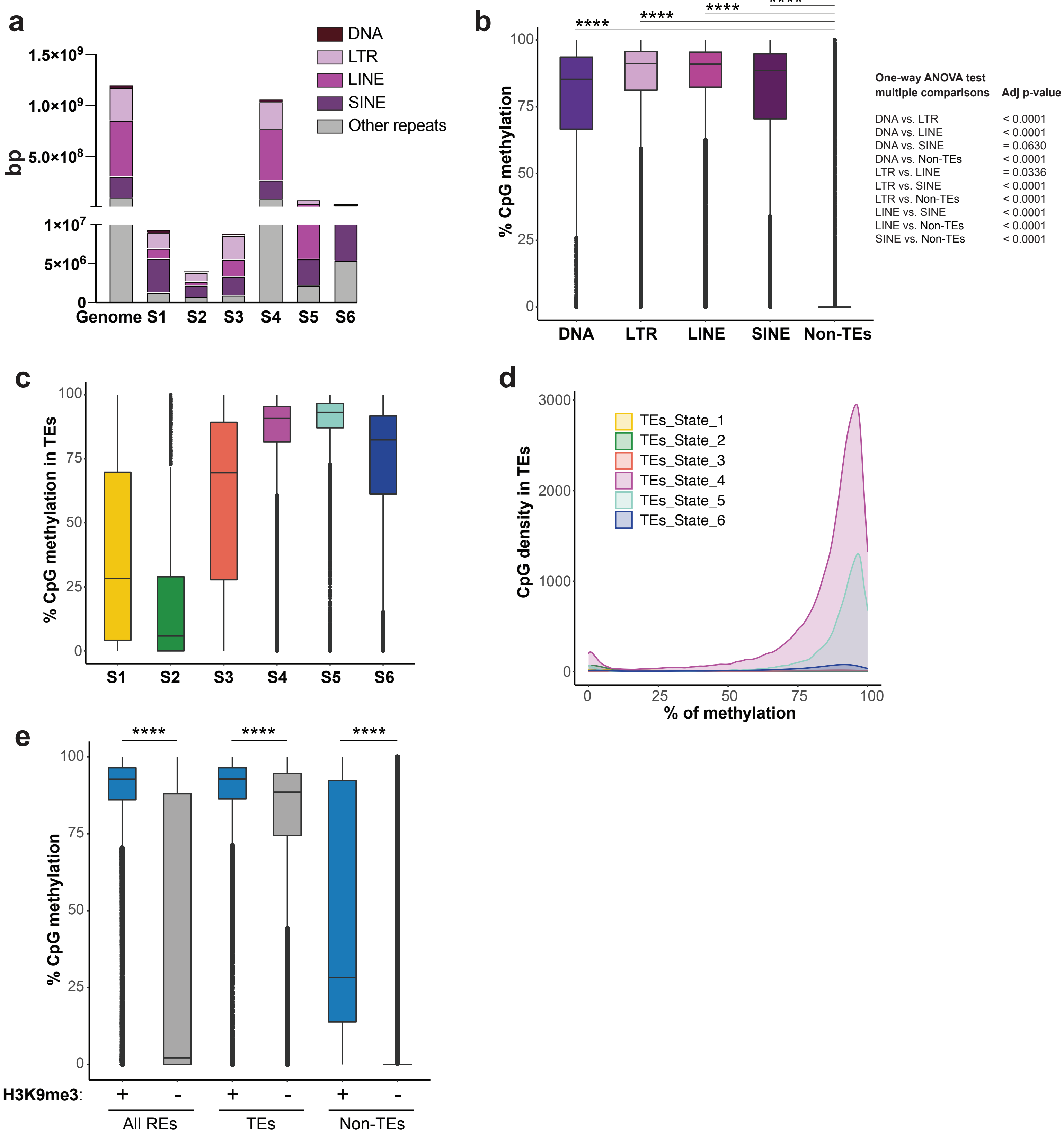
a
DNA
LTR
LINE
SINE
Other repeats
bp
1.5×10^9
1.0×10^9
5.0×10^8
1×10^7
5×10^6
0
Genome S1 S2 S3 S4 S5 S6
b
****
****
****
****
% CpG methylation
100
75
50
25
0
DNA LTR LINE SINE Non-TEs
One-way ANOVA test multiple comparisons
Adj p-value
DNA vs. LTR < 0.0001
DNA vs. LINE < 0.0001
DNA vs. SINE = 0.0630
DNA vs. Non-TEs < 0.0001
LTR vs. LINE = 0.0336
LTR vs. SINE < 0.0001
LTR vs. Non-TEs < 0.0001
LINE vs. SINE < 0.0001
LINE vs. Non-TEs < 0.0001
SINE vs. Non-TEs < 0.0001
c
% CpG methylation in TEs
100
75
50
25
0
S1 S2 S3 S4 S5 S6
d
CpG density in TEs
3000
2000
1000
0
TEs_State_1
TEs_State_2
TEs_State_3
TEs_State_4
TEs_State_5
TEs_State_6
0 25 50 75 100
% of methylation
e
****
****
****
% CpG methylation
100
75
50
25
0
H3K9me3: + - + - + -
All REs
TEs
Non-TEs

Supplementary Figure 3. DNA methylation, H3K9me3 and H3K27me3 occupancy of transposable elements in the mouse liver.

a. TEs annotation of mm10 reference genome and in each chromatin state.

b. Percentage of DNA methylation across TE subclasses (DNA, LTR, SINE and LINE and other repetitive elements) shows that TEs are more methylated compared to other repetitive elements. p-values were calculated by one way ANOVA adjusted with a Tukey multiple comparison test. Adjusted p-values are indicated on the figure. **c.** Percentage of DNA methylation of TE subclasses in each state showing that TEs present in S4 and S5 are more methylated compared to the ones in other states. **d**. Density plot of CpGs detected in the eRRBS data in TEs divided by each chromatin state shows that the vast majority of CpGs are in S4 and S5 and they are highly methylated. A small fraction of CpGs is present in S1, S2, S3 and S6. **e.** Box plot of DNA methylation of repetitive elements occupied by H3K9me3 shows that the TEs occupied by H3K9me3 are more methylated than TEs without H3K9me3. Two-sided p-values are calculated by unpaired non-parametric Mann-Whitney test. **** means $p\text{-value} < 0.0001$. Data in **b-c-e** are represented as box-and-whisker plots where center lines are the medians, the lower and upper hinges correspond to the first and third quartiles, the whiskers extend from the hinges respectively to the largest or smallest values no further than $1.5 \times \text{IQR}$ (inter-quartile range), while data beyond the end of the whiskers are outlying points that are plotted individually.

Suppl. Fig4

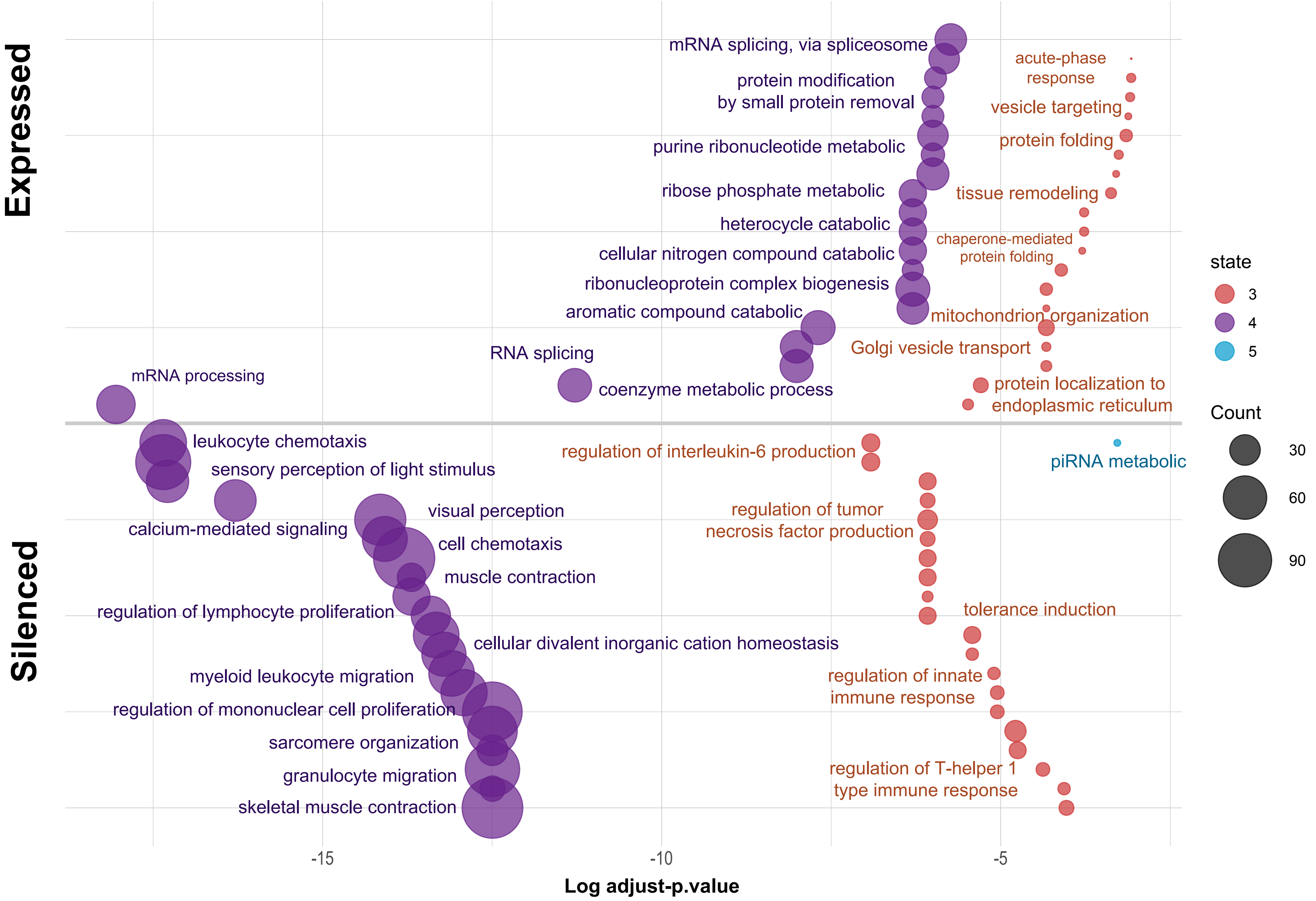

Expressed
Silenced
mRNA splicing, via spliceosome
protein modification
by small protein removal
purine ribonucleotide metabolic
ribose phosphate metabolic
heterocycle catabolic
cellular nitrogen compound catabolic
ribonucleoprotein complex biogenesis
aromatic compound catabolic
RNA splicing
mRNA processing
coenzyme metabolic process
acute-phase
response
vesicle targeting
protein folding
tissue remodeling
chaperone-mediated
protein folding
mitochondrion organization
Golgi vesicle transport
protein localization to
endoplasmic reticulum
leukocyte chemotaxis
sensory perception of light stimulus
calcium-mediated signaling
visual perception
cell chemotaxis
muscle contraction
regulation of lymphocyte proliferation
cellular divalent inorganic cation homeostasis
myeloid leukocyte migration
regulation of mononuclear cell proliferation
sarcomere organization
granulocyte migration
skeletal muscle contraction
regulation of interleukin-6 production
regulation of tumor
necrosis factor production
tolerance induction
regulation of innate
immune response
regulation of T-helper 1
type immune response
piRNA metabolic
state
3
4
5
Count
30
60
90
-15
-10
-5
Log adjust-p.value

Supplementary Figure 4. Gene functions are segregated into distinct chromatin states. Significant GO pathways represented as expressed and silenced in S3, S4 and S5. The bubble color and size correspond to state and gene number, respectively. Note that state 5 has only 132 genes and of these, only a few are grouped by function.

Suppl. Fig5

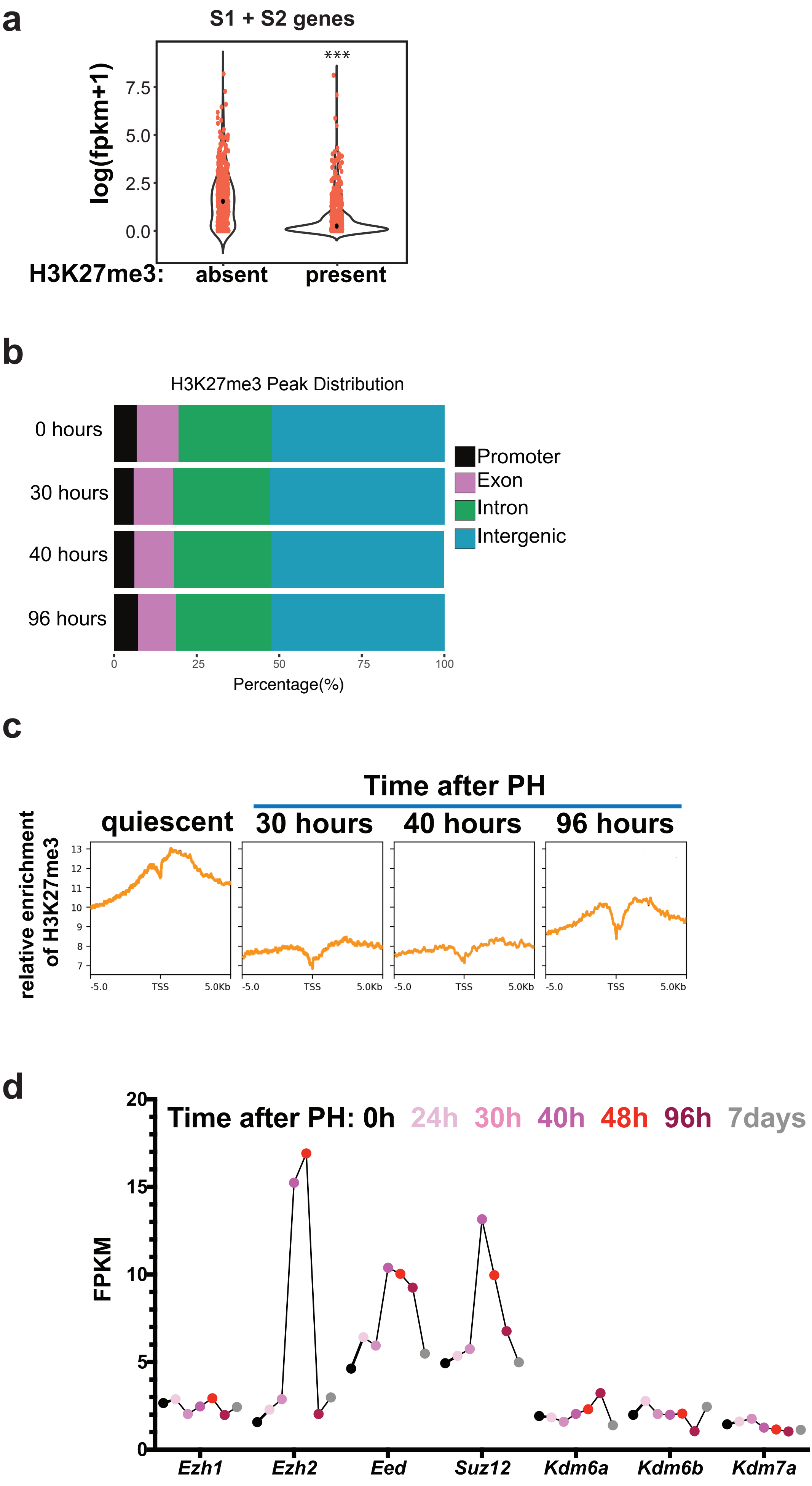

a
S1 + S2 genes
log(fpkm+1)
7.5
5.0
2.5
0.0
***
H3K27me3: absent present
b
H3K27me3 Peak Distribution
0 hours
30 hours
40 hours
96 hours
Promoter
Exon
Intron
Intergenic
0 25 50 75 100
Percentage(%)
c
Time after PH
quiescent
30 hours
40 hours
96 hours
relative enrichment of H3K27me3
13
12
11
10
9
8
7
-5.0 TSS 5.0Kb
d
Time after PH: 0h 24h 30h 40h 48h 96h 7days
FPKM
20
15
10
5
0
Ezh1
Ezh2
Eed
Suz12
Kdm6a
Kdm6b
Kdm7a

Supplementary Figure 5. H3K27me3 redistribution across the hepatic genome during liver regeneration.

a. Expression of genes from S1 and S2 categorized based on presence or absence of H3K27me3 occupancy at the TSS. Genes marked with H3K27me3 are significantly lower than those without; Welch t-test was performed on log transformed fpkm in R, *** indicates p-value < 2.2e-16 by two-side t-test.

b. Annotation of genomic elements occupied by H3K27me3 peaks at 4 time points after PH.

c. H3K27me3 enrichment profiling across the TSS of all genes in quiescent and regenerating livers.

d. FPKM values of H3K27me3 methyltransferases and demethylases after PH based on RNAseq analysis.

Suppl. Fig6

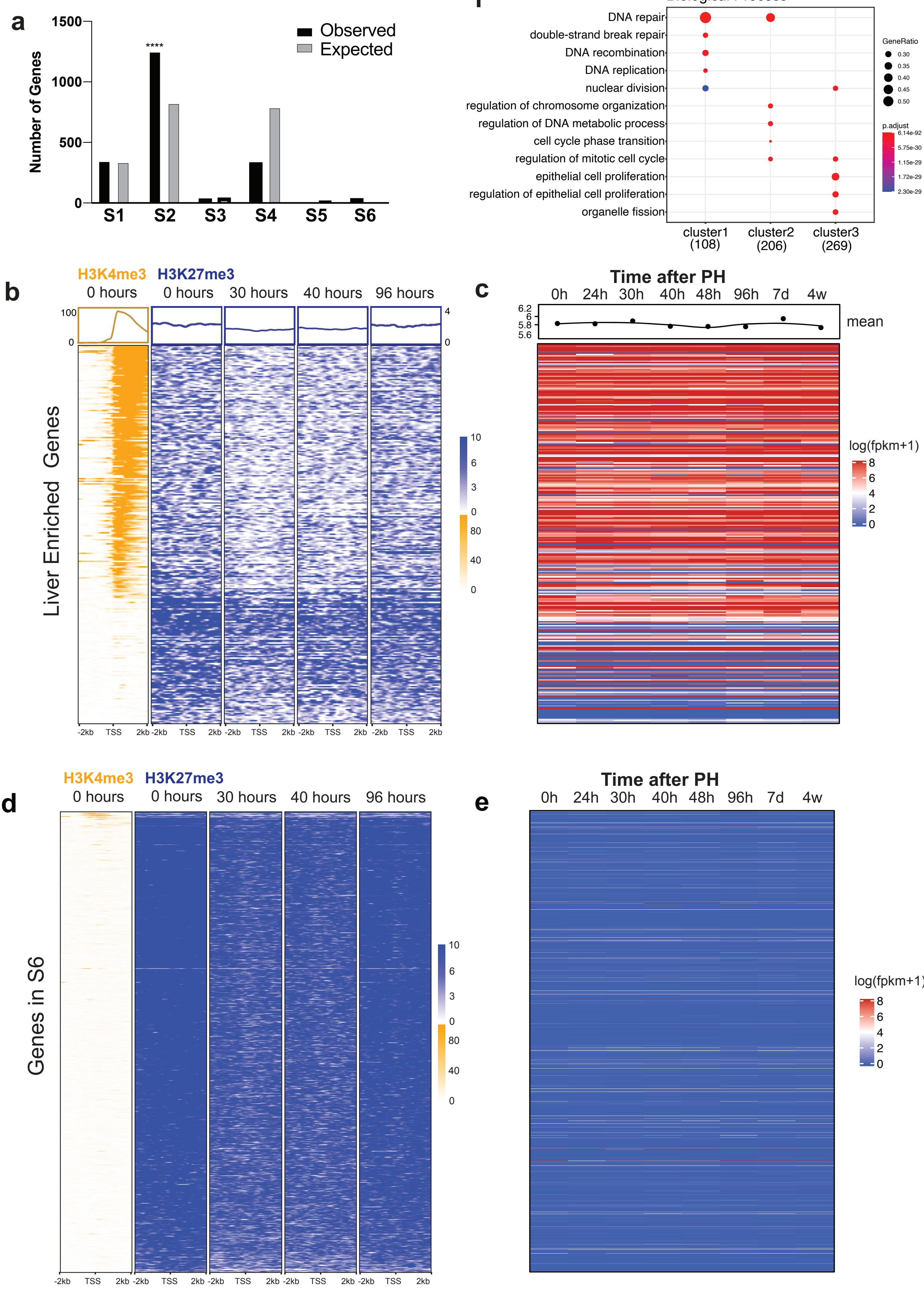
a
Number of Genes
1500
1000
500
0
****
Observed
Expected
S1
S2
S3
S4
S5
S6
f
Biological Process
DNA repair
double-strand break repair
DNA recombination
DNA replication
nuclear division
regulation of chromosome organization
regulation of DNA metabolic process
cell cycle phase transition
regulation of mitotic cell cycle
epithelial cell proliferation
regulation of epithelial cell proliferation
organelle fission
cluster1 (108)
cluster2 (206)
cluster3 (269)
GeneRatio
0.30
0.35
0.40
0.45
0.50
p.adjust
6.14e-92
5.75e-30
1.15e-29
1.72e-29
2.30e-29
b
H3K4me3
H3K27me3
0 hours
0 hours
30 hours
40 hours
96 hours
Liver Enriched Genes
-2kb
TSS
2kb
c
Time after PH
0h
24h
30h
40h
48h
96h
7d
4w
mean
log(fpkm+1)
d
Genes in S6
e

Supplementary Figure 6. Expression of genes from states and functional category during regeneration.

a. Actual numbers of expressed genes verses expected number of genes calculated from the total genes in each state.

b. H3K4me3 and H3K27me3 occupancy across the TSS of liver enriched genes in quiescent livers and H3K27me3 occupancy at 0, 30, 40 and 96 hours after PH.

c. Heatmap of liver enriched genes showing that they are consistently expressing during regeneration.

d. H3K4me3 and H3K27me3 occupancy across the TSS of all genes in S6 at 0 and H3K27me3 occupancy at 0, 30, 40 and 96 hours after PH.

e. Heatmap of all genes in S6 shows that they are not re-expressed during regeneration.

f. significant GO terms in 3 clusters, top 5 GO categories that don’t include all clusters are shown.

Suppl. Fig7

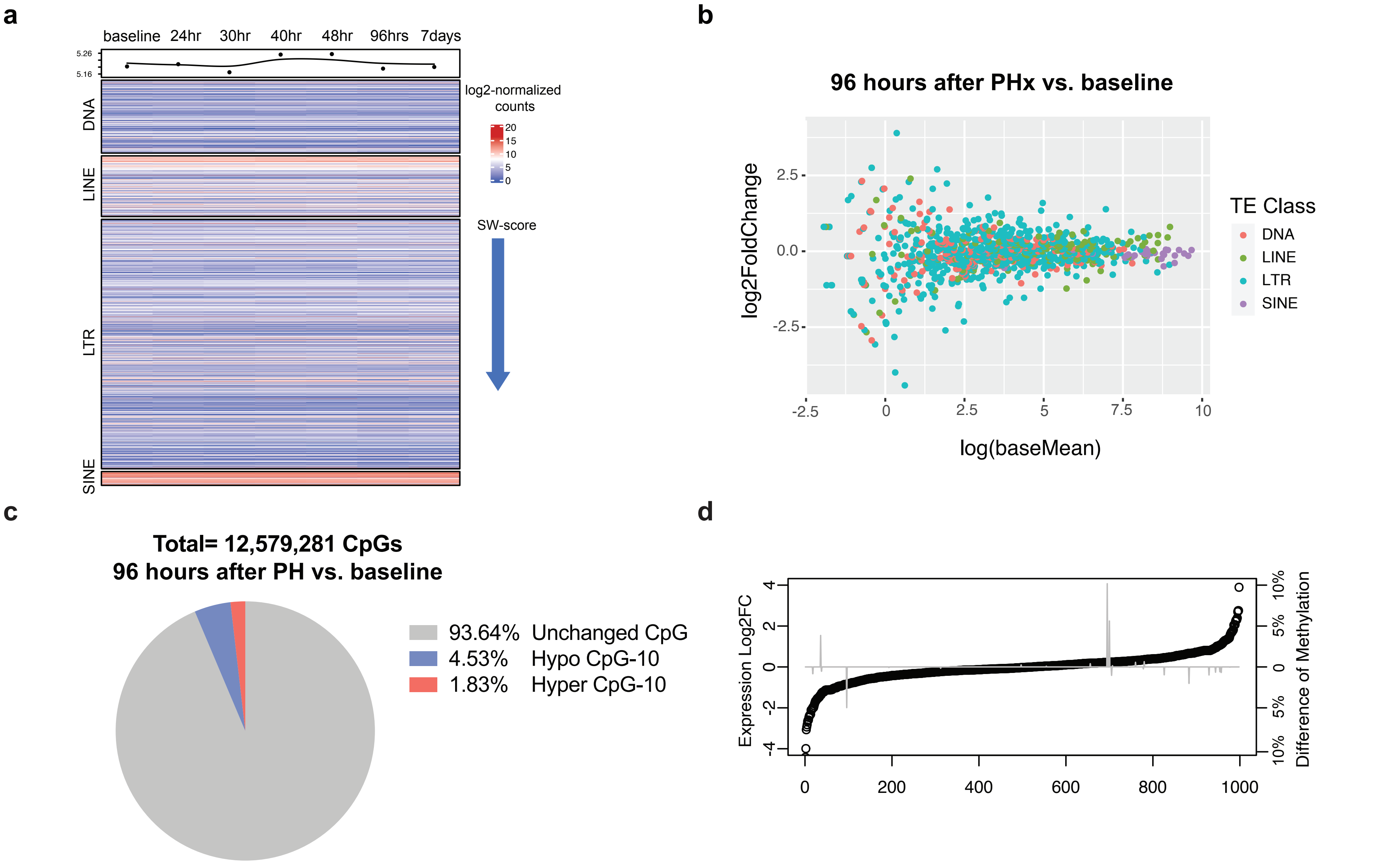

Supplementary Figure 7. TE expression during liver regeneration and correlation with DNA methylation.
a. Expression heatmap of annotated TEs at 7 time points during liver regeneration, categorized into classes and rank ordered by age (sw-score).
b. MAplot showing differential expression of TEs segregated by class at comparing 0 and 96 hours after PH.
c. The difference of CpG methylation between 96 hours after PH and 0.
d. Integration of TE expression change and TE methylation change, log2 transformed expression FC of 96 compared to 0 aligning with difference of methylation.

Suppl. Fig8

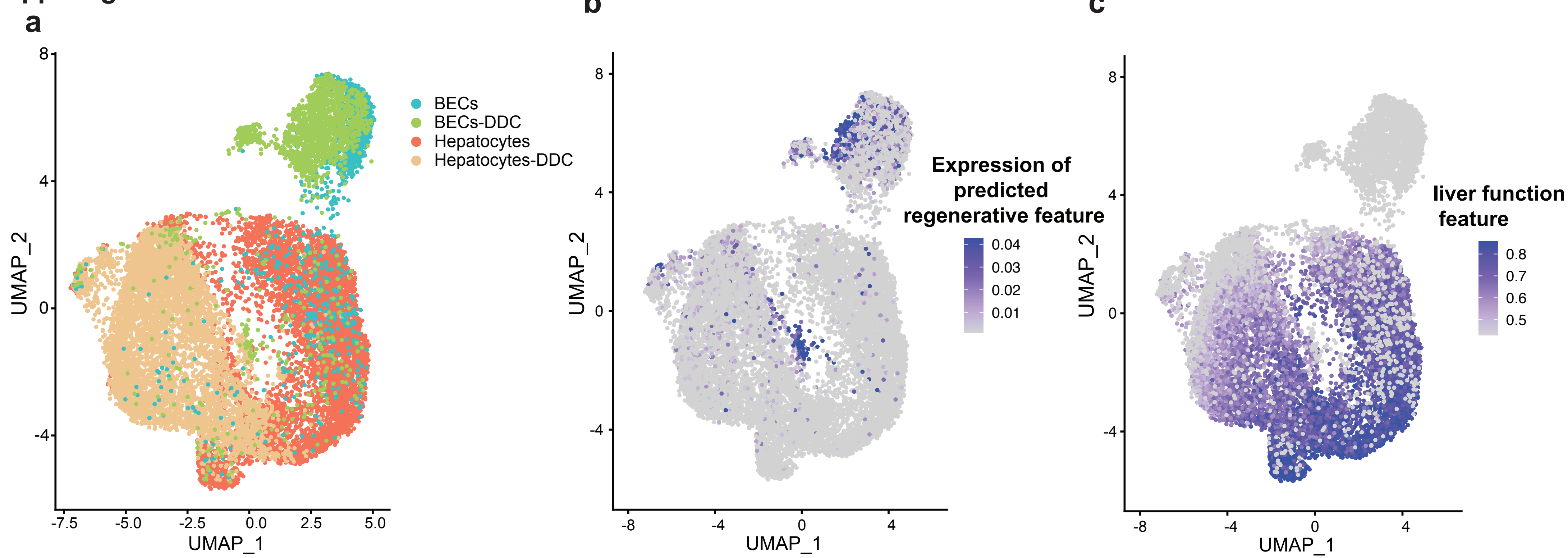

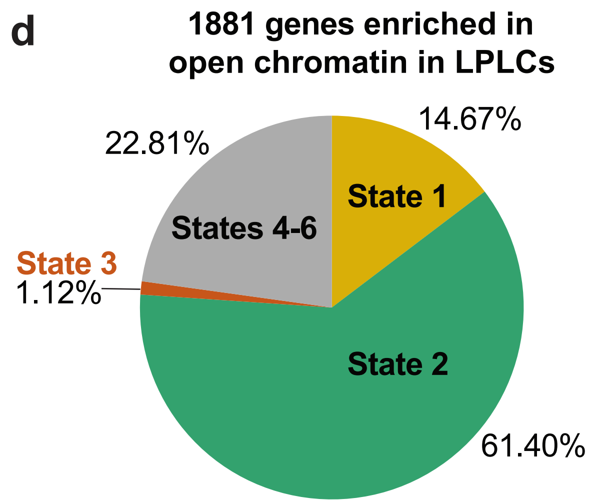

Supplementary Figure 8. Heterogeneity of regenerative potentials response to liver injury.

a. Uniform Manifold Approximation and Projection (UMAP) was used as a dimension reduction tool to visualize the complex connections among the cells. Distribution of healthy hepatocytes and biliary epithelial cells (BECs), injury hepatocytes and BECs are shown in the UMAP as colored by each cell type.

b. Expression level of the regenerative gene feature which compresses the expression levels of all the genes examined in Figure 5E into a single value and are plotted on the same UMAP reduction.

c. Expression level of the liver function gene feature in the same UMAP reduction

d. Distribution of the 1881 genes enriched in open chromatin status from liver progenitor like cells (LPLC) falling into open chromatin states S1, S2 and S3.

Table S1 Sequencing data set summary

Sample Name	Reads	Align Rate	Library Prep	Called Peaks	GEO Accession
baseline WT ATAC	63,811,006.00	97.49%	PE	23,943	GSM4634005
baseline WT_b_H2az	61,446,619.00	99.45%	SE	99,226	GSM4634007
baseline WT H3K9me3 (round 2)	124,010,776.00	90.53%	PE	123,180	GSM3753284
baseline WT H3K4me3 (round 2)	60,208,280.00	99.28%	PE	35,334	GSM3753286
Regenerating WT H3K27me3 - 0hr (round 3)	82,659,374.00	93.87%	PE	343,983	GSM3753289
Regenerating WT H3K27me3 - 30hr (round 3)	67,682,622.00	99.97%	PE	340,301	GSM3753290
Regenerating WT H3K27me3 - 40hr (round 3)	63,809,278.00	99.96%	PE	274,886	GSM3753291
Regenerating WT H3K27me3 - 96hr (round 3)	77,672,450.00	99.96%	PE	255,970	GSM3753292
