# Supplementary material for: Chromatin states shaped by an epigenetic code confer regenerative potential to the mouse liver
Source: Nat Commun. 2021 Jul 5;12:4110. doi: 10.1038/s41467-021-24466-1 (PMC8257577; doi:10.1038/s41467-021-24466-1)
Supplement: Supplementary file 3 — Description of Additional Supplementary Files' [file 41467_2021_24466_MOESM3_ESM.docx]

**Description of Additional Supplementary Files**

Zhang et al.

Description: Sequencing data set summary
File Name: Supplementary Table1_DataSummary

Description: Genes encompassed by each chromatin state. The presence of H3K27me3 is noted on each gene in S1, S2 and S6.
File Name: Supplementary Dataset1_StateGenes

Description: Lists of genes that are segregated by functions relevant to liver regeneration.

Liver function genes are plotted in Figure 4C as red dots. Cell Cycle genes are clustered in Figure 5C.
File Name: Supplementary Dataset2_FunctionalGene

Description: Genes in chromatin states are categorized in distinct ontologies. All genes in each state were categorized as expressed or silenced, and each category was then analyzed for GO of biological function. Significantly enriched GO categories are shown for each state. GO categories relevant to liver regeneration are italicized and were used for subsequent analysis in Figure 5.
File Name: Supplementary Dataset3_FullGO

Description: Raw images of western plots
File Name: Source Data
