# Supplementary material for: Chromatin states shaped by an epigenetic code confer regenerative potential to the mouse liver
Source: Nat Commun. 2021 Jul 5;12:4110. doi: 10.1038/s41467-021-24466-1 (PMC8257577; doi:10.1038/s41467-021-24466-1)
Supplement: Supplementary file 7 — Reporting Summary [file 41467_2021_24466_MOESM7_ESM.pdf]

nature research

Corresponding author(s): NCOMMS-20-37054A

Last updated by author(s): 2021-5-22

Reporting Summary

Nature Research wishes to improve the reproducibility of the work that we publish. This form provides structure for consistency and transparency in reporting. For further information on Nature Research policies, see our Editorial Policies and the Editorial Policy Checklist.

Statistics

For all statistical analyses, confirm that the following items are present in the figure legend, table legend, main text, or Methods section.

n/a	Confirmed	
☐	☒	The exact sample size (n) for each experimental group/condition, given as a discrete number and unit of measurement
☐	☒	A statement on whether measurements were taken from distinct samples or whether the same sample was measured repeatedly
☐	☒	The statistical test(s) used AND whether they are one- or two-sided<br>*Only common tests should be described solely by name; describe more complex techniques in the Methods section.*
☒	☐	A description of all covariates tested
☐	☒	A description of any assumptions or corrections, such as tests of normality and adjustment for multiple comparisons
☐	☒	A full description of the statistical parameters including central tendency (e.g. means) or other basic estimates (e.g. regression coefficient) AND variation (e.g. standard deviation) or associated estimates of uncertainty (e.g. confidence intervals)
☐	☒	For null hypothesis testing, the test statistic (e.g. F, t, r) with confidence intervals, effect sizes, degrees of freedom and P value noted<br>*Give P values as exact values whenever suitable.*
☒	☐	For Bayesian analysis, information on the choice of priors and Markov chain Monte Carlo settings
☒	☐	For hierarchical and complex designs, identification of the appropriate level for tests and full reporting of outcomes
☒	☐	Estimates of effect sizes (e.g. Cohen's d, Pearson's r), indicating how they were calculated

Our web collection on statistics for biologists contains articles on many of the points above.

Software and code

Policy information about availability of computer code

Data collection	no software was used for data collection
Data analysis	RNA-seq: tophat2 v2.1.0, Cufflinks v2.2.1, HTSeq<br>Chip-seq and ATAC-seq: BWA-MEM, PICARD 2.20.8, macs2 2.1., deeptools 2.5.1<br>RRBS: Bismark-0.22.3<br>ChromHMM v1.21 was used for chromatin states prediction<br>R 3.6.1 was used for data processing and visualization

For manuscripts utilizing custom algorithms or software that are central to the research but not yet described in published literature, software must be made available to editors and reviewers. We strongly encourage code deposition in a community repository (e.g. GitHub). See the Nature Research guidelines for submitting code & software for further information.

Data

Policy information about availability of data

All manuscripts must include a data availability statement. This statement should provide the following information, where applicable:

- Accession codes, unique identifiers, or web links for publicly available datasets
- A list of figures that have associated raw data
- A description of any restrictions on data availability

RNA-seq: GSE125008, Chip-seq: GSE125006 , ATAC-seq: GSE153090, Encode data is available at the GEO repository: GSE31039
Figure 1A, Suppl. Fig 1, Suppl. Fig 2 and Suppl. Table 1 are associated with raw data.
All GEO repositories are publicly available.

Field-specific reporting

Please select the one below that is the best fit for your research. If you are not sure, read the appropriate sections before making your selection.

☒ Life sciences ☐ Behavioural & social sciences ☐ Ecological, evolutionary & environmental sciences

For a reference copy of the document with all sections, see nature.com/documents/nr-reporting-summary-flat.pdf

Life sciences study design

All studies must disclose on these points even when the disclosure is negative.

Sample size	Sample size was evaluated according to the estimated variance by experienced investigators; in some cases cost considerations influenced sample size
Data exclusions	Clustering based on the all gene expression from RNA-seq, those that were not clustered within the condition are treated as outliers.
Replication	Replicates in groups are checked and validated by clustering based on measurements.
Randomization	Animals were allocated randomly, while sample collection and measurements were conducted without bias.
Blinding	To track the time-course, complete blinding is impossible and therefore investigators were diligent about adhering to he same procedures and keeping the same standard for sample collection and analysis.

Reporting for specific materials, systems and methods

We require information from authors about some types of materials, experimental systems and methods used in many studies. Here, indicate whether each material, system or method listed is relevant to your study. If you are not sure if a list item applies to your research, read the appropriate section before selecting a response.

Materials & experimental systems

n/a	Involved in the study	
☐	☒	Antibodies
☒	☐	Eukaryotic cell lines
☒	☐	Palaeontology and archaeology
☐	☒	Animals and other organisms
☒	☐	Human research participants
☒	☐	Clinical data
☒	☐	Dual use research of concern

Methods

n/a	Involved in the study	
☐	☒	ChIP-seq
☒	☐	Flow cytometry
☒	☐	MRI-based neuroimaging

Antibodies

Antibodies used	Antibodies used are anti-H3K4me3 (Abcam, ab1012), anti-H3K27me3 (Active Motif, 61017), anti-H3K9me3 (Active Motif, 39161) and anti-H2AZ (Abcam, ab4174).
Validation	Each antibody was validated by the manufacturer and the H2AZ and H3K27 antibody was validated in our lab using Western Blotting.

Animals and other organisms

Policy information about studies involving animals; ARRIVE guidelines recommended for reporting animal research

Laboratory animals	Wild type male mice on a congenic C57Bl/6 background between 6-8 weeks of age
Wild animals	*Provide details on animals observed in or captured in the field; report species, sex and age where possible. Describe how animals were caught and transported and what happened to captive animals after the study (if killed, explain why and describe method; if released, say where and when) OR state that the study did not involve wild animals.*
Field-collected samples	Temperature, humidity, and light:dark cycles were controlled and mice were fed food and water ad libitum.
Ethics oversight	Icahn School of Medicine at Mount Sinai and NYU Abu Dhabi Institutional Animal Care and Use Committee (IUCUC)

Note that full information on the approval of the study protocol must also be provided in the manuscript.

ChIP-seq

Data deposition

☒ Confirm that both raw and final processed data have been deposited in a public database such as GEO.

☒ Confirm that you have deposited or provided access to graph files (e.g. BED files) for the called peaks.

Data access links
May remain private before publication.

https://www.ncbi.nlm.nih.gov/geo/query/acc.cgi?acc=GSE125006
https://www.ncbi.nlm.nih.gov/geo/query/acc.cgi?acc=GSE153090

Files in database submission

called peaks files and raw files

Genome browser session
(e.g. UCSC)

http://topaz.abudhabi.nyu.edu/epigenome/?genome=mm10&session=nkqDVsJKRu&statusId=603324232

Methodology

Replicates

single samples were used for all the ChIPseq data

Sequencing depth

Sample Name	Reads	Align Rate	Library Prep
baseline WT_H2az	61,446,619	99.45%	SE
baseline WT H3K9me3,	124,010,776	90.53%	PE
baseline WT H3K4me3,	60,208,280	99.28%	PE
baseline H3K27me3 - 0hr,	82,659,374	93.87%	PE
PHx WT H3K27me3 - 30hr,	67,682,622	99.97%	PE
PHx WT H3K27me3 - 40hr,	63,809,278	99.96%	PE
PHx WT H3K27me3 - 96hr,	77,672,450	99.96%	PE

Antibodies

Antibodies used are anti-H3K4me3 (Abcam, ab1012), anti-H3K27me3 (Active Motif, 61017), anti-H3K9me3 (Active Motif, 39161) and anti-H2AZ (Abcam, ab4174).

Peak calling parameters

```
# Alignment
bwa mem -t 12 -M {$Ref} {$sample}.read1.fastq.gz {$sample}.read2.fastq.gz > {$sample}_aligned.sam
samtools view -Su {$sample}_aligned.sam > {$sample}_aligned.bam
samtools sort -@ 12 -o {$sample}_sorted.bam {$sample}_aligned.bam
The reference genome {$Ref} is GRCm38.p4
# Peak calling
Macs2 was used for peak calling, specific parameters are as below:
H3K4me3 -g mm -p 0.01 --keep-dup 1
H2AZ -g mm -p 0.01 --keep-dup 1
H3K27me3 -g mm -p 0.01 --broad --broad-cutoff 0.05 --keep-dup 1
H3K27me3 -g mm -p 0.01 --broad --broad-cutoff 0.01 --keep-dup 1
# convert bam files to bigwig files
bamCoverage -b {$sample}_sorted.bam -o {$sample}.bigwig -of bigwig -bs 30 --smoothLength 300 --extendReads 200 --
normalizeUsingRPKM
```

Data quality

Sample Name	Peak numbers
baseline WT_H2az	99,226
baseline WT H3K9me3,	123,180
baseline WT H3K4me3,	35,334
baseline H3K27me3 - 0hr,	343,983
PHx WT H3K27me3 - 30hr,	340,301
PHx WT H3K27me3 - 40hr,	274,886
PHx WT H3K27me3 - 96hr,	255,970

Software

Illumina Casava1.8 software used for basecalling; The raw reads were quality assessed using FastQC v0.11.5, then quality trimmed using Trimmomatic. were "trimmomatic_adapter.fa:2:30:10 TRAILING:3 LEADING:3 SLIDINGWINDOW:4:15 MINLEN:36"; Reads that passed quality control were aligned to reference genome GRCm38.p4 using bwa-mem; The resulting alignments were then sorted and indexed with samtools; Peaks calling was generated by MAC2, parameters are described as above.
